# Exploration of experience and sustained engagement with a web-based cognitive rehabilitation intervention amongst patients with aggressive lymphoma: a qualitative sub-study

**DOI:** 10.1007/s00520-026-10928-z

**Published:** 2026-06-24

**Authors:** Priscilla Gates, Sian Carlon-Garbutt, Haryana M. Dhillon, Heather J. Green, Karla Gough, Michael Dickinson, Tracey Dryden, Jade Guarnera, Karen Caeyenberghs

**Affiliations:** 1 School of Psychology, Deakin University, Burwood, VIC Australia; 2 Centre for Health Services Research in Cancer, Peter MacCallum Cancer Centre, Parkville, VIC Australia; 3 Sir Peter MacCallum, Department of Oncology, The University of Melbourne, Parkville, VIC Australia; 4 Psycho-Oncology Cooperative Research Group, The University of Sydney, Sydney, NSW Australia; 5 Griffith University, Gold Coast, QLD Australia; 6 Department of Nursing, The University of Melbourne, Parkville, VIC Australia; 7 Clinical Haematology, Peter MacCallum Cancer Centre and Royal Melbourne Hospital, Parkville, VIC Australia

**Keywords:** Cancer-related cognitive impairment, Cognitive rehabilitation intervention, Aggressive lymphoma, Qualitative study

## Abstract

**Background:**

Cancer-related cognitive impairment (CRCI), a common side effect of cancer and its treatment, is characterised by difficulties in memory, attention and executive function. This qualitative sub-study was part of a single-site, parallel-group, pilot randomised controlled trial in which recruitment and retention exceeded our expectations. The aim was to explore participants’ experience and motivation for sustained engagement with a web-based cognitive rehabilitation (eReCog) intervention amongst people with aggressive lymphoma who were self-reporting cognitive decline.

**Methods:**

We used an inductive qualitative approach, conducting semi-structured interviews with fourteen participants. Interviews were recorded, transcribed and a reflexive thematic approach was used to describe and interpret key themes and sub-themes in the data.

**Results:**

Fourteen interviews were completed. We extracted four themes describing participants experience and motivation for sustained engagement with eReCog. These included information needs, experience of participation, support and ease of use. Participants were motivated to engage to gain knowledge and strategies to manage their CRCI symptoms; they enjoyed the experience and felt validated via the online community created. Finally, they valued the additional support they received and appreciated the convenience and flexibility of the web-based program.

**Conclusions:**

Our findings show that engagement with eReCog was driven by perceived cognitive improvements, psychosocial benefits and accessibility. Addressing both cognitive and psychosocial needs is warranted in web-based rehabilitation to foster continued participation engagement. Web-based cognitive rehabilitation interventions should enhance accessibility and earlier integration into the cancer trajectory to optimise long-term survivorship care in people with haematological cancers should be considered.

**Trial registration:**

Australian New Zealand Clinical Trials Registry ACTRN 12623000705684 on 30th June 2023.

## Introduction

Cancer-related cognitive impairment (CRCI) is a disabling side-effect of cancer and its treatment characterised by impaired memory, attention and executive functioning [1, 2]. While CRCI is well documented in solid tumour populations, such as those with breast cancer [3, 4], evidence indicates that people with haematological malignancies, including those with aggressive lymphoma, experience comparable patterns of impairment [5–7]. For example, survivors of aggressive lymphoma have demonstrated worse performance on measures of memory, executive function and processing speed compared to healthy controls [8, 9]. These cognitive difficulties can precede treatment, with up to 30% exhibiting impairments at diagnosis, up to 75% during treatment, and can persist in as many as 35% after treatment completion [10]. Given the relatively young age at diagnosis and high survival rates, many long-term survivors of Hodgkin lymphoma contend with enduring cognitive sequelae across critical life stages and are more likely to be unemployed or receiving a disability pension [11]. Similar trends have been reported in mixed-cancer cohorts, with cognitive symptoms a primary reason for reduced working hours and early retirement [12].

While no pharmacological treatments to date have demonstrated efficacy [1, 13], behavioural interventions, particularly cognitive rehabilitation, have emerged as the most promising approach [14, 15]. Cognitive rehabilitation typically involves a combination of psychoeducation, metacognitive strategy training and cognitive exercises targeting memory, attention and executive function [15]. Several systematic reviews and meta-analyses across oncology populations have demonstrated that cognitive rehabilitation interventions can produce improvements in both subjective and objective cognitive outcomes [13, 15]. Yang et al. synthesised 46 randomised controlled trials in breast cancer survivors and found that cognitive rehabilitation ranked highest for improving memory and attention, with similar gains in perceived cognition observed across mixed cancer cohorts [16]. Despite promising findings, the evidence base for cognitive rehabilitation remains disproportionately focused on breast cancer populations, with only 13% of cognitive rehabilitation studies including people who were diagnosed with lymphoma [15].

Moreover, many traditional cognitive rehabilitation programmes are resource-intensive, require repeated in-person attendance and place additional demands on survivors already managing fatigue, work reintegration and emotional adjustment [13]. These practical barriers have prompted interest in alternative delivery formats, particularly web-based cognitive rehabilitation. Unlike in-person formats, web-based delivery offers flexibility, giving equitable access to underserved populations and those living in rural areas [17].

Qualitative research suggests the value of cognitive rehabilitation extends beyond cognitive improvement, offering survivors a way to reclaim agency, reshape identity and process the experience of cancer treatment [4]. In an interpretative phenomenological study, Joyce et al. found that mixed cancer populations engaging in a cognitive rehabilitation program described a renewed sense of belonging, relief at being understood and a reconnection with their capabilities.^4^ Similarly, Mayo et al. evaluated the feasibility of a computerised cognitive training program in people who had completed treatment for a haematological cancer, and found that, although adherence was limited by symptom burden and competing demands, participants valued structure, accountability and feedback [18].

A limitation of the evaluation of digital health interventions, however, is high attrition. App-based programs for chronic disease often face substantial challenges in sustaining participant engagement over time [19]. In a systematic review and meta-analysis, Meyerowitz-Katz et al. reported an average dropout rate of 43% across 17 trials of app-based interventions [19]. These challenges are also evident in cancer survivorship research, with attrition rates in web-based cancer cognitive rehabilitation studies up to 40% [20]. In contrast, Mihuta and Green’s evaluation of a web-based cognitive rehabilitation program, Responding to Cognitive Concerns (eReCog), reported attrition rates of 8–16% suggesting relatively strong engagement among breast cancer survivors [20].

This qualitative sub-study was undertaken as part of a larger single-site, parallel-group, pilot randomised controlled trial (RCT) conducted in the clinical haematology service at the Peter MacCallum Cancer Centre in Melbourne, Australia. The pilot RCT investigated the feasibility, acceptability and preliminary efficacy of eReCog among people treated for aggressive lymphoma who were self-reporting CRCI [21] [22]. Perceived reduction in cognitive functioning was based on the single‐item Cognitive Change Screen (i.e. a score of 28.5 or higher) shown to have sensitivity and specificity in people with mixed cancer diagnoses [23]. Participants were randomised to either eReCog or usual care, with the program showing feasibility, acceptability and potential efficacy [22]. Recruitment and retention to the pilot RCT exceeded our expectations, with 38 of 53 eligible individuals (72%) enrolled within 10 months, and 36 of these 38 participants (95%) completing the trial. This included completion of eReCog as well as study assessments including neuropsychological tests and patient‐reported outcome measures [PROMs] administered at baseline and 8-week follow‐up [22]. These unexpectedly high recruitment and retention rates served as the rationale for undertaking this qualitative sub-study, which aimed to examine how participants experienced and sustained their engagement with eReCog.

## Methods

### Study design

This sub-study implemented an exploratory design taking an inductive approach to data collection and analysis, recognising that meaning is constructed from the data.

PG (PhD) is a Nursing Research Fellow and an experienced haematology advanced practice nurse, with expertise in haematological cancer survivorship, which initiated her interest in CRCI as a clinical problem.

### Intervention

The eReCog program is grounded in cognitive behavioural therapy principles and is designed to provide psychoeducation on cognitive impairment associated with cancer and its treatment, alongside practical strategies for improving memory, attention and self-regulation [20–22]. The program comprised four weekly online modules focused on (a) ageing, health, cancer and cognitive function; (b) memory; (c) attention and (d) fatigue, emotions and cognition. Each module contained 13–20 interactive activities (e.g. goal setting, memory and attention exercises, strategy application and self-care) which take approximately 30–60 min to complete. Participants are expected to complete one module per week across 4 weeks. Additional features include de-identified online interactive pages where reflection exercises, asynchronous group discussion and weekly online homework tasks were available. It was supported by a programme facilitator via email exchange, who tracked progress through the modules to ensure participants completed each module before access to the next was enabled.[20–22, 24].

### Sampling and data collection

Pilot trial participants were 18 years or older and had completed chemotherapy for aggressive lymphoma within the past 5 years and were in remission [21]. Pilot trial participants who had completed eReCog were invited to attend an interview for the sub-study. Written informed consent was obtained as part of the pilot RCT, and reconsent to participate in the interview was recorded at the start of each interview. Semi-structured interviews were conducted via telephone at a time convenient for participants and were conducted by PG. Interviews continued to data saturation (three consecutive interviews with no themes or concepts arising [25]. Interviews were semi-structured with open-ended questions about participants’ experiences of the eReCog programme, factors that supported or hindered motivation, and recommendations for future delivery. The complete list of questions is provided in Table 1. All interviews were audio-recorded and transcripts generated using Trint software [26]. Transcripts were subsequently checked for accuracy against the original audio recordings. Following quality assessment, all transcripts were de-identified ahead of data analysis.

**Table 1:** Semi-structured interview questions

No	Question
1	What cognitive changes prompted you to agree to participate in the study?
2	Did you have any expectations of the cognitive rehabilitation study at that time—that is, was there something in particular that made you decide/agree to take part?
3	If you had any expectations that led you to take part—were they met?
4	What has kept you motivated to participate in the study?
5	Were there some positive things about the cognitive rehabilitation program?
6	Were there some negative things about the cognitive rehabilitation program?
7	Do you have any recommendations for anything we could have done differently in the study?

### Data analysis

Interview transcripts were analysed using reflexive thematic analysis, a flexible and rigorous method for identifying, analysing and reporting patterns within qualitative data [27] [28]. Braun and Clarke’s six-phase approach was followed, whereby we (a) familiarised ourselves with the data through repeated reading of transcripts; (b) generated initial codes by systematically coding interesting features across the dataset; (c) searched for themes and subthemes by collating related codes and mapping patterns and divergences across participants; (d) reviewed themes against the dataset to ensure they were accurate reflections of the data; (e) defined and named themes by writing concise analytic summaries articulating each theme’s central organising concept and any subthemes; these were used to capture distinct, nuanced aspects within broader themes [29] and (f) produced a report by integrating thematic insights with illustrative quotes. Coding was conducted manually by two independent researchers (PG, SCG). Discrepancies were resolved through discussion and consensus, and a third researcher (KC) was consulted on a weekly basis. Analytic reflexivity was supported through four peer-debriefing meetings in which the team examined assumptions, tested alternative readings and refined the coding framework and thematic map. Thematic analysis was particularly suited to this study’s aim of exploring subjective experiences in depth, while allowing flexibility to accommodate diverse narratives across patients.

## Results

Eighteen participants completed eReCog. All but one participant who had died were invited to take part in the sub-study, 14 agreed. Two participants lacked interest, and one had relapsed.

### Sample characteristics

Sample characteristics are summarised in Table 2. The median age was 44 years (range 29–61), and seven participants were female (50%). Median education was 15 years (range 12–18 years) and most participants had Hodgkin lymphoma (71%).

**Table 2:** Demographic and clinical characteristics (*n* = 14)

	*n* (*%*) or *Mdn* (range)
**Age (years)**	44 (range = 29–61)
**Sex**	
Male	7 (50)
Female	7 (50)
**Marital status**	
Married/de facto	13 (93)
Single	1 (7)
**Education (years)**	15 (range = 12–18)
**Lymphoma subtype**	
DLBCL	3 (21)
HL	10 (71)
Other	1 (7)

Abbreviations: *Mdn* median, *HL* Hodgkin lymphoma, *DLBCL* diffuse large B-cell lymphoma. Percentages may not total 100 due to rounding

### Qualitative insights

Fourteen interviews were conducted. Date saturation was achieved at this point as no additional themes or insights emerged. The interviews were conducted on average 14 months (range = 9–19) after completion of eReCog and lasted an average of 16 min (range: 11–20).

#### Themes

**Fig. 1:**
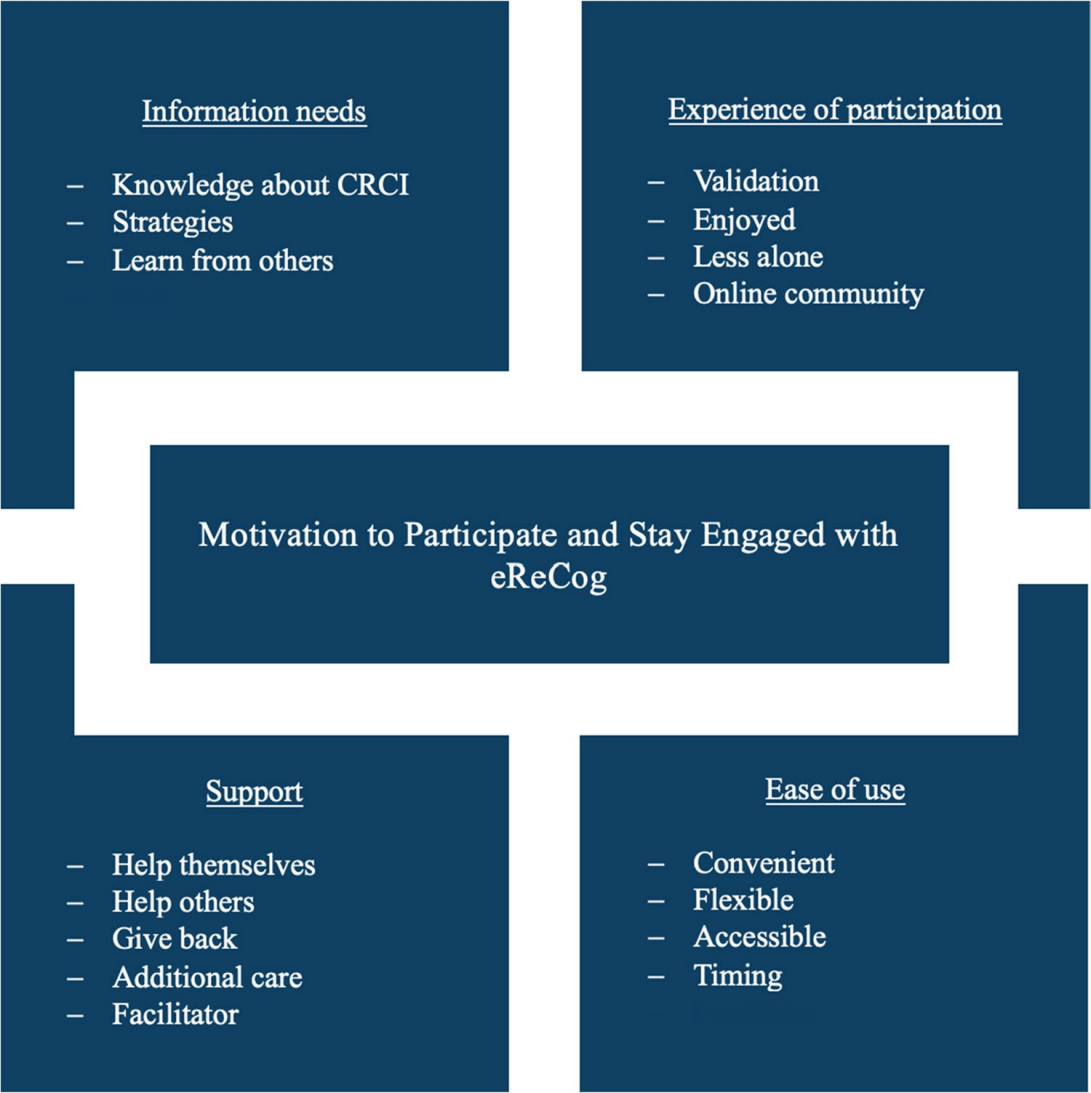
Overview of themes and subthemes

Four overarching themes were derived from the data: (1) information needs; (2) support; (3) ease of use and (4) experience of participation. These broad themes included subthemes (see Fig. 1).

## Theme one: information needs

Participants described a strong desire to better understand their experiences of CRCI. Many expressed feelings of confusion and frustration when trying to make sense of memory lapses, concentration difficulties and poor clarity of thought. Several participants described the disruptive impact of CRCI on their daily functioning, such as not being able to recall conversations or respond to questions as quickly as before.

Brain fog, that’s the main one… I couldn’t answer questions that people were asking me… I couldn’t answer them straight away. I needed to think about it, and I was like, very foggy. It was like I was saying the answer in my brain, but it wasn’t actually coming out of my mouth (p2)

People have told me that they told me things before, and I just didn’t remember. Sometimes I remember part of the conversation, but not the whole lot. (p12)

Engaging with eReCog was seen as an opportunity to gain clarity and reassurance. Participants described being motivated to participate in the programme with the hope of learning strategies to address these difficulties.

I thought this (eReCog) could probably help my memory or brain fog. I thought, maybe there’s some sort of exercises or there’s some sort of technique I could use to improve (p2)

Several reflected on how eReCog supported improvements in their memory and understanding and felt they were able to apply the specific techniques and strategies they learnt.

I felt every week my memory was progressing… And I was getting more understanding and getting more knowledge and getting… what I had asked for. (p13)

I think there was one module where you had to think of… something to try and remember more things you know, and it sort of made sense, and I enjoyed it. I still use that technique to this day. (p5)

Learning from others also emerged as a positive influence as the anonymous discussion forum embedded within eReCog allowed participants to compare experiences and gain insight from others navigating similar challenges.

I liked the activity and being able to see other people's responses that I could go through. When I didn't understand some of the responses I would Google if I wanted to know more information about a particular subject… that led me to another website with like lymphoma survivors and going through their stories. (p9)

Participants also valued the learnings they received that were in addition to activities that directly assessed memory and thinking. Many commented on the support they received for their mental health, particularly the benefits of the meditation exercise.

The meditation I felt very useful. You know, when I had some free time, I would turn that on and listen to it. I still have it to this day. (p13)

## Theme two: Experience of participation

Engagement with eReCog was described as an enjoyable and positive experience, which participants felt was integral to their sustained engagement with the programme.

I think the whole process was like a really good experience. I think it was a great experience. (p3)

I did enjoy it. I wouldn’t have done that if I didn’t enjoy it. (p1)

This sense of enjoyment underpinned participants’ motivation and reinforced their overall positive experience of participation. In addition, reading other participants’ anonymous responses on the forum normalised the challenges of CRCI and reduced feelings of experiencing these challenges in isolation. For many participants, this created a sense that their cognitive difficulties were shared rather than personal failings.

Like being able to read other people’s responses and hearing that they were also going through their own side effects… it reassured me that this is normal. Like this is an okay reaction to have. (p14)

That, you know, the other participants’ responses and things… yeah, that creates a bit of a community. (p6)

## Theme three: support

Participants described the opportunity to receive enhanced support regarding current concerns as central for motivating both their initial participation and sustained engagement with eReCog. The program was often viewed as a form of additional care that complemented their standard of care, by offering support in areas that participants felt were not typically addressed. While their treating teams were largely focused on monitoring disease response and managing the physical side effects of chemotherapy, participants felt eReCog provided additional support responsive to their cognitive and emotional experiences.

It gave me a lot of different resources… the meditation, for example, and the modules helped provide a very good approach around what was happening. (p9)

Participants also highlighted the role of the online facilitator as particularly valuable in keeping them engaged and motivated. Having someone to check in, guide progress, and provide encouragement gave participants a sense of accountability and was reassuring.

I think there was someone… who would email me just to check in. It was a reminder, you know, I know you haven’t done it yet, but you know, just if you could get it done this week… so that sort of made it easier and exceptionally punctual. (p6)

Alongside this external support, participants also described how eReCog created an opportunity to support themselves. For many, the program instilled a sense of responsibility and discipline at a time when self-motivation was difficult to sustain.

It gave me a responsibility that I had to do these things… I needed support with doing things for myself and that was good for me. (p11)

In addition, many participants were motivated by the opportunity to support others. Many described a strong sense of altruism, noting their participation was a way to contribute to improving the care of future patients who would face similar challenges.

I was so grateful that I survived. I wanted to be part of studies so I could help other people in the future (p11)

Some participants attributed their motivation to give back to the hospital, where they had completed their cancer treatment.

I think that’s more my gratitude for the hospital what made me take part (p4)

## Theme four: ease of use

The accessibility of eReCog was central to engagement with the programme. Participants emphasised the value of being able to access the programme online, at a place, time and pace that suited them. This flexibility was described as critical in supporting participation while managing competing demands during their recovery.

It wasn’t something I had to do in the morning or anything like that. It was just when I was relaxed and had nothing else to focus on, and that was good. Because then I could answer the questions properly as well, so there was no rush or pressure. (p14)

I like online because I can look at it whenever I want. (p9)

The ability to complete modules at their own pace, and to revisit content when needed, was also valued.

You can do it in your own time. You didn’t have to show up and do something. If you were tight for time, you could do it the next night. (p5)

The timing of delivery of eReCog was also highlighted. Some participants felt the program may have been even more beneficial if delivered earlier after their chemotherapy ended.

I think soon after treatment would be good… like after your last chemo and when life sort of starts again, when you start adjusting back to life. (p3)

In contrast, others reflected that engaging too early in the treatment process would have been too overwhelming:

wouldn’t have mentally been able to focus on it. (p1)

Participants also raised concerns about the accessibility of the content, noting that some aspects of the programme design and language might be more easily navigated by individuals with higher levels of education.

It depends on the background of the person who's doing it… it's easier when you've had some sort of university or higher education involvement that you find something easier to understand. (p1)

The layout or the delivery of it, I think it was a bit clunky… a little bit wordy… sometimes I had to reread things, to try and make them sink in a little bit (p7)

Some participants further suggested that extending the program beyond 4 weeks may have been beneficial.

It would be good if it went for a little bit longer and I probably would have still continued to engage in it. (p6)

Despite these reflections, no participant could identify other topics or resources that could have been included to enhance or extend the programme when asked.

## Discussion

This qualitative sub-study explored participants’ experience and sustained engagement with eReCog amongst people who had completed chemotherapy for aggressive lymphoma. Through thematic analysis, we identified four key themes: (a) information needs; (b) experience of participation; (c) support and (d) ease of use. These themes represent factors that shaped participants’ experience and motivation for sustained engagement within the programme. 

Previous interventional studies in cancer survivors frequently report low recruitment rates and substantial barriers to participation, particularly due to practical barriers such as time commitment due to longitudinal designs. Digital interventions often face considerable attrition and disengagement, with an average dropout rate of 43% [19] [30]. In contrast, recruitment (72% of eligible participants recruited over 10 months) and retention (95% of enrolled participants on trial at follow-up) to our eReCog pilot were high and exceeded our expectations [31].

Our findings show that a key motivation for participation and sustained engagement with eReCog was the desire to better understand CRCI. Participants frequently described feeling confused, frustrated and uncertain around memory lapses and brain fog, echoing prior studies showing that these symptoms are often experienced as unpredictable and invalidating [2, 32], and persist following chemotherapy for aggressive lymphoma [8].

Our results suggest that unmet informational needs regarding CRCI persist well after treatment completion, aligning with evidence that psychoeducation on CRCI is rarely integrated into standard follow-up care [2, 32]. Participants highlighted the importance of acquiring practical strategies to manage cognitive concerns, as these not only alleviated symptoms but fostered sustained motivation. This is consistent with other studies which highlight that strategy-based rehabilitation enhances adherence when survivors are provided with tools they perceive as immediately useful [15].

Engagement with eReCog was also reinforced by the broader positive experience of participation. Participants benefitted from the peer discussion forum, where reading others’ anonymous responses reassured them that their cognitive difficulties were shared by others. In other words, sharing experiences through the peer forum fostered a sense of community that helped normalise their challenges. This finding resonates with previous qualitative research in cancer survivors demonstrating the perceived value of cognitive rehabilitation often extends beyond improvements in memory or attention to encompass feelings of connection, belonging and relief at being understood [4]. This is consistent with a study in young adult survivors of childhood cancer who valued the discussion forum within a web-based intervention because it fostered a sense of belonging with peers [33]. Similarly, survivors of haematological cancer identified peer support as a critical facilitator of adherence to home-based cognitive training, with the absence of opportunities for peer interaction described as a barrier to sustained participation [18].

Participants emphasised support as central to sustaining motivation and adherence with the eReCog programme. Participants viewed the study as a way of giving back, either to their treating hospital or to support future patients. Altruistic motives have been consistently reported in cancer survivorship research, with Gates et al. (2022) reporting participants were motivated to remain engaged in CRCI research, because it offered an opportunity to help others and to contribute to scientific progress [34]. Similarly, in a survey of 249 cancer survivors, 92% identified altruism and a commitment to help others as key drivers to participating in clinical trials [35]. Participants also valued the regular online contact with the facilitator, and received on average 17 emails from the facilitator providing support, reassurance, accountability and encouragement to complete the eReCog modules [22]. This email contact is similar to other studies using eReCog where an average of 21 emails was sent per participant [20]. This aligns with growing evidence that digital health interventions are more effective when human support is integrated, resulting in lower rates of attrition [19]. Formalised accountability has been shown to be critical for sustained motivation with home-based computerised cognitive training programme for survivors of haematological cancer [18].

The majority of participants appreciated and valued the ease of use of eReCog. The flexibility of the program allowed them to complete eReCog amid competing demands of their recovery, such as being able to complete modules at their own pace, in their own home, and revisit content when needed. This supports that accessibility itself can act as an active mechanism of engagement, which aligns with digital health models ascribing convenience as a determinant of adherence [30]. Our findings are consistent with previous studies showing that accessibility enhances engagement in digital rehabilitation, leading to high satisfaction and modest cognitive benefits [36, 37].

### Clinical implications

These findings reinforce the value of digital cognitive rehabilitation in meeting the information and emotional needs of people after chemotherapy for aggressive lymphoma, with peer connection and facilitator support particularly important for sustaining engagement. Our sample was highly educated (*Mdn* = 15 years of education). To meet the needs of people with shorter durations of formal education, it may be useful to incorporate plain language principles, shorter and less text-dense modules, and greater use of pictograms and videos to reinforce key concepts within eReCog. Previous studies show that simplifying language in online materials improves self-efficacy [38], which may in turn increase usability and long-term adherence for people with lower levels of education. [39]

Several participants indicated they would have remained engaged with the eReCog program even if the duration was longer than four weeks. A recent systematic review of cognitive rehabilitation programs for adult cancer survivors showed a high degree of heterogeneity in terms of duration of the intervention ranging from 4 to 12 weeks [40]. Another review in a non-cancer population suggests that cognitive interventions of longer duration (≥ 30 min), delivered less frequently but over several months, are associated with higher cognitive benefits [41]. However, these gains must be balanced against participant burden, which may result in higher attrition. Participants also indicated that eReCog may have been more valuable if offered earlier (i.e. during or soon after chemotherapy finished). This is consistent with previous work demonstrating that cognitive impairments are present in people with aggressive lymphoma prior to chemotherapy [9, 10], so earlier delivery may increase both clinical impact and perceived relevance.

Finally, many participants valued the mediation exercise embedded in eReCog which assisted them in coping with their frustration associated with their CRCI symptoms. Studies show mindfulness meditation can improve attention, working memory and executive functioning [42], and reduce subjective cognitive complaints in people experiencing CRCI [43].

### Limitations

Several limitations should be considered when interpreting the findings of this study. First, on average, interviews were conducted 14 months (range 9–19 months) after participants completed eReCog. This gap may have introduced recall bias, leading to inaccurate or incomplete recollection. However, delayed follow-up can also be advantageous, as it may provide insight into the longer-term benefits and sustainability of the intervention effect.

Second, the study focused on a single cancer disease group, which may narrow the scope of applicability to cancer populations more broadly. Nevertheless, it represents one of the first qualitative investigations of cognitive rehabilitation in survivors of aggressive lymphoma. Related to this, our sample comprised equal gender representation, addressing the imbalance observed in previous web-based CRCI rehabilitation studies that disproportionately enrol women with breast cancer.

Finally, participants were generally well-educated, which may limit the generalisability of findings to others with lower literacy levels. As digital interventions rely on users’ literacy, inequities in this domain may undermine engagement and outcomes. While this is a limitation, it also highlighted challenges that can potentially guide refinement of eReCog in future trials.

## Conclusion

Our study highlights participants’ excellent experience and engagement with a web-based cognitive rehabilitation programme among people who have completed chemotherapy for aggressive lymphoma. Participants’ accounts revealed that engagement was sustained not only by the programme’s educational and strategy-based content, but by the experience it offered, the sense of support and altruism fostered, and the ease of use of its online delivery. This study contributes novel insights into how a cognitive rehabilitation programme can potentially be delivered to meet the needs of people with haematological cancer, supporting the delivery of equitable digital interventions to improve cognitive outcomes and quality of life.

## Data Availability

De-identified data supporting the findings of this study are available from the corresponding author upon request.

## Code availability

NA.
